# In memoriam: Mark Wainberg, PhD (1945–2017)

**DOI:** 10.7448/IAS.20.1.22206

**Published:** 2017-07-13

**Authors:** Papa Salif Sow, Susan Kippax, Marlène Bras, Kenneth H Mayer, Linda-Gail Bekker, Chris Beyrer

**Affiliations:** ^a^ Department of Infectious Diseases, University of Dakar, Dakar, Senegal; ^b^ Journal of the International AIDS Society, Geneva, Switzerland; ^c^ Social Policy Research Centre, University of New South Wales, Sydney, Australia; ^d^ The Fenway Institute, Fenway Health, Boston, MA, USA; ^e^ Desmond Tutu HIV Research Center, University of Cape Town, Cape Town, South Africa; ^f^ Center for Public Health and Human Rights, Johns Hopkins University, Baltimore, MD, USA

“When I look back on my career, I always feel that the most important contribution of my life was political and not scientific” – Mark Wainberg

It is with a heavy heart that the scientific community learned of the passing of Mark Wainberg, who died at the age of 71 in Bal Harbour, Florida. At the time of his death, Mark was a lead investigator at the Lady Davis Institute for Medical Research at the Jewish General Hospital, the director of the McGill University AIDS Centre and an Editor-in-Chief of the *Journal of the International AIDS Society* (JIAS).

Mark devoted his life to AIDS research, and his work has contributed directly to the saving of millions of lives around the planet. In 1989, Mark’s team characterized the antiviral activity of 3TC or lamivudine, which played a fundamental role in curving the HIV epidemic.

Mark served as the president of the International AIDS Society (IAS) between 1998 and 2000. Hoping to draw media attention and to achieve political commitment to the lack of access to antiretroviral drugs in developing countries, Mark oversaw the first conference to take place in South Africa in 2000: the 13th International AIDS Conference. In Durban, alongside Nelson Mandela, he urged African governments and international institutions to become more involved in the response to the epidemic: an historic moment in HIV in Africa and the world. The AIDS 2000 Conference became a turning point in the history of HIV and AIDS as it led a global movement to bring life-saving HIV treatment to developing countries, leading to the first United Nations Declaration on HIV/AIDS from 2001 and the creation of The Global Fund to Fight AIDS, Tuberculosis and Malaria and PEPFAR.

In 2004, Mark Wainberg pioneered the idea for a new journal devoted to HIV and AIDS: the *Journal of the International AIDS Society* (JIAS). In the years that followed, he was an outstanding Editor-in-Chief, enthusiastic, committed and passionate. Mark was central to the journal’s growing success and he was instrumental in ensuring that social, behavioural, political and economic research was included alongside biomedical and clinical research. The JIAS is one of the very few international HV/AIDS journals to do so. The publication of the impact factor of JIAS was always a much awaited moment by Mark. For JIAS, this impact factor continues to increase, from 3.256 in 2012 to 6.296 in 2016: an outcome for which Mark deserves much applause and appreciation.

As well as wanting the JIAS to be a first-class journal, Mark also envisioned that the journal would enable young and inexperienced researchers, especially from resource-limited settings, to publish their biomedical, epidemiological, and social and political science, health economics and operational research. To this end with the support of the JIAS, Mark participated on a regular basis in the organization of skills-building workshops on scientific writing and publishing at international conferences. These workshops were often organized during the major international conferences of the IAS, with a significant participation of young African and other researchers from middle- and low-income countries.

Mark was also co-organizer of INTEREST Workshop, whose main objective was to enable African researchers to present their research and remain continually up to date on research developments and best practices. He did his best to travel to Africa each year and participate in INTEREST meetings, contributing to the training and capacity building of young African researchers. Mark received many African microbiologists in his laboratory at McGill University with a Canadian Cooperation grant for their Master and PhD training. These researchers on their return home continued to benefit from the scientific and technical support of Mark and his team; they significantly contributed to patient management in terms of microbiology and virology (viral load and resistance monitoring).

Mark will always be remembered as a brilliant scientist whose research contributed in saving the lives of people living with HIV, but first and foremost as a fierce advocate for access to treatment and human rights giving a voice to the voiceless. More than ever, political commitment to the HIV response is a critical issue and Mark’s true legacy will be the new generation of researchers he worked with and inspired, and who will keep up the momentum to end HIV.

He will be sorely missed.

